# Diastolic dysfunction is common and predicts outcome after cardiac surgery

**DOI:** 10.1186/s13019-018-0744-3

**Published:** 2018-06-15

**Authors:** Thomas S. Metkus, Alejandro Suarez-Pierre, Todd C. Crawford, Jennifer S. Lawton, Lee Goeddel, Jeffrey Dodd-o, Monica Mukherjee, Theodore P. Abraham, Glenn J. Whitman

**Affiliations:** 1 Division of Cardiology, Johns Hopkins University School of Medicine, 600 N. Wolfe Street, Blalock 524 D2, Baltimore, MD 21287 USA; 2 Division of Cardiac Surgery, Johns Hopkins University School of Medicine, Baltimore, MD USA; 3 Department of Anesthesia and Critical Care Medicine, Johns Hopkins University School of Medicine, Baltimore, MD USA; 4 Division of Cardiology, Department of Medicine, University of California, San Francisco, 505 Parnassus Ave., Suite M344 San Francisco, San Francisco, CA USA

**Keywords:** CABG, AVR, Echocardiography, Diastolic dysfunction, Mechanical ventilation

## Abstract

**Background:**

Diastolic dysfunction (DD) identified on echocardiography predicts mortality after cardiac surgery, however the most useful diastolic parameters for assessment and the association of DD with prolonged mechanical ventilation, ICU re-admission, and hospital length of stay are not established.

**Methods:**

We included patients that underwent coronary artery bypass grafting (CABG), aortic valve replacement (AVR) or a combined procedure (CAB-AVR) from 2010 to 2016, and who had preoperative transthoracic echocardiography (TTE) at our institution within 6 months of the operation. Diastolic function was graded using the transmitral E and A waves and the septal tissue Doppler velocity. We performed logistic regression to assess the association of grade of DD with a composite endpoint of death, prolonged mechanical ventilation, ICU readmission during hospitalization, and hospital length of stay longer than 14 days.

**Results:**

Between 2010 and 2016, 577 patients were eligible for inclusion. DD was common, with 42% of the cohort manifesting grade II or grade III DD. Rates of death and prolonged ventilation increased across grades of DD and across quartiles of increasing LV filling pressure, assessed by the E/e’ ratio. Adjusting for age, sex, procedure, systolic and diastolic function, both systolic (odds ratio 0.68 95% CI 0.55–0.85 per inter-quartile increase in LVEF) and diastolic function (odds ratio 1.31 95% CI 1.04–1.66 per increasing DD grade) both independently predicted outcome.

**Conclusion:**

Diastolic dysfunction is common among patients undergoing cardiac surgery and is associated with death, prolonged mechanical ventilation, and prolonged hospital and ICU length of stay independent of systolic dysfunction.

## Background

Diastolic dysfunction (DD) consists of abnormalities in myocardial relaxation and increased left ventricular stiffness leading to elevated cardiac filling pressures and, in the extreme form, decreased stroke volume [1, 2]. DD is a cardinal manifestation of heart failure with preserved ejection fraction (HFpEF) [3] and is impacted by volume status [4], positive pressure ventilation [5–7], and revascularization [8]. Diastolic function assessed by echocardiography has been shown to be associated with atrial fibrillation after cardiac surgery in some [9] but not all [10] studies, and various echocardiographic markers of diastolic function may predict outcomes including death, major morbidity, and difficulty separating from cardiopulmonary bypass [11–17]. The specific diastolic parameters most useful for predicting outcomes after cardiac surgery are not clear, and the association of DD with important outcomes including prolonged need for mechanical ventilation, intensive care unit re-admission and prolonged length of stay have not been established.

The purpose of this study was to assess the association of pre-operative diastolic function assessed by echocardiography with post-operative outcomes following coronary artery bypass grafting (CABG), aortic valve replacement (AVR) or combined CABG and AVR. We hypothesized that a higher grade of DD would be associated with an increased risk of postoperative mortality, prolonged hospital length of stay, and need for prolonged mechanical ventilation after surgery.

## Methods

We performed a retrospective study to determine the association of echocardiographic DD with outcomes after CAB, AVR, or CAB-AVR. We hypothesized that higher grade of DD assessed using the transmitral E and A waves and the septal tissue Doppler velocity (e’) and higher cardiac filling pressures estimated by the ratio of E to e’ velocity would both be associated with higher risk of postoperative mortality, prolonged hospital length of stay, and need for prolonged mechanical ventilation after surgery. The study was approved by the Johns Hopkins Hospital institutional review board.

### Patient population

Between 2010 and 2016, 2596 patients underwent CAB, 809 patients underwent AVR, and 386 underwent CAB-AVR. The present study included patients undergoing isolated CAB, isolated AVR and combined CAB-AVR between July 2010 and July 2016 who had a full transthoracic echocardiogram at our institution within the 6 months prior to surgery, who were in sinus rhythm pre-operatively, and had measurable mitral inflow pattern, systolic function, and tissue Doppler as described below. Using these criteria, 577 patients were included in the current study.

### Echocardiography

We extracted data from echocardiography reports for eligible patients. Studies were performed by certified sonographers and interpreting physicians were expert practicing echocardiographers at the Johns Hopkins Hospital. Diastolic function was assessed using transmitral diastolic flow profile including peak early diastolic velocity (E wave velocity) and peak late diastolic filling velocity (A wave velocity) [1, 2]. We assessed septal annular velocity (e’) using Tissue Doppler Imaging (TDI) consistent with recommended guidelines [1, 2]. The ratio of E to e’ velocity was assessed [1, 2] as a surrogate for LV filling pressure [18]. The choice of parameters used to assess diastolic function was made on the basis of guideline-recommended approaches and anticipated feasibility of obtaining measurements on both transthoracic and intra-operative trans-esophageal echocardiography. Using the mitral inflow patterns from the 2016 guidelines on echocardiographic assessment of diastolic function [2], we graded diastolic function as follows:

- Grade 0: normal left ventricular ejection fraction, e’ > 7 cm/s and E/e’ < 14
- Grade I: E/A < 0.9 and E < 50 cm/s
- Grade I: E/A < 0.8 with E > 50 cm/s or 0.8 < E/A < 2 AND E/e’ < 14
- Grade II: E/A < 0.8 with E > 50 cm/s or 0.8 < E/A < 2 AND E/e’ > 14
- Grade III: E/A >  2

### Statistics

Demographic and clinical characteristics were compared across grades of diastolic function using the Kruskal-Wallis test for continuous variables and Pearson’s chi-squared test for categorical variables. We report the percentage of patients suffering death, prolonged mechanical ventilation or both across grades of diastolic function and across quartiles of E/e’ ratio. We performed logistic regression to assess the association of grade of DD with a composite endpoint of events of a priori clinical interest: death within 30 days, prolonged mechanical ventilation, ICU readmission during hospitalization, and hospital length of stay longer than 14 days. Univariate models and models which adjusted for clinical factors of a priori clinical interest were performed. Covariates included age, sex, procedure (CABG v. AVR v. CAB-AVR), and systolic function. A two-tailed *P* value less than 0.05 was considered statistically significant. All analyses were performed using StataSE version 14.0 (StataCorp Inc., College Station, TX).

## Results

Between 2010 and 2016, 577 patients were eligible for inclusion. The specific procedures included CABG alone in 77%, AVR alone in 16% and a combined procedure in 6%. A median of 4 (interquartile range (IQR) 1.5–10) days passed between the transthoracic echocardiogram used to grade diastolic function and the operation. DD was common, with 42% of the cohort manifesting grade II or grade III DD compared to 58% of the group with grade 0 or grade I. Most patients had preserved systolic function, with median LVEF 55% (IQR 45–60%).

Demographic and clinical characteristics are shown in the Table 1 for the entire cohort and across grades of diastolic function. Patients with more advanced DD were older, had higher body-mass index, more concomitant systolic dysfunction, had a higher prevalence of known heart failure, diabetes, and elevated creatinine.

**Table 1:** Demographic and clinical characteristics and outcomes of the cohort and across grades of diastolic function

Diastolic grade (N)	Total (577)	Grade 0 (155)	Grade 1 (179)	Grade 2 (203)	Grade 3 (40)	*P*
Age (years)	66 (58–74)	62 (53–69)	66 (58–75)	70 (60–78)	69.5 (61–78.5)	0.0001
Timing of echo pre-op (days)	4 (1.5–10)	2.5 (1–8)	3 (1.4–6.3)	5 (2–26)	7.1 (2.3–13.2)	0.0001
Female sex	160 (27.7%)	25 (16%)	46 (26%)	80 (39%)	9 (23%)	0.0001
Height (cm)	172.7 (165–179)	175.3 (167.6–180.3)	172.7 (165–180.3)	170.2 (162.6–177.8)	172.7 (162.6–179.1)	0.0003
Weight (kg)	84.8 (74.5–98.1)	85.3 (74–96)	83.9 (74.5–94.4)	86 (75.2–103.3)	84.3 (72.5–102)	0.4
Body-mass index (kg/m2)	28.6 (25.5–32.6)	27.7 (25.3–31.2)	28.3 (25.2–31.9)	29.9 (26.5–35.0)	29.4 (25.4–32.8)	0.001
E (cm/s)	80.5 (64.2–98.7)	76.9 (66–90.8)	62.8 (51.4–72)	96.7 (82.2–112)	115.5 (96.4–134)	0.0001
A (cm/s)	79.5 (64.4–97.2)	71.4 (61.2–87.1)	78.5 (65.8–89.3)	95.4 (75.5–116)	39.9 (32.2–54.6)	0.0001
Decel time (ms)	222 (188–269)	220 (187–259)	235 (202–288)	226 (191–272)	156 (130–191.5)	0.0001
LVEF (%)	55 (45–60)	60 (55–65)	55 (45–60)	55 (40–60)	30 (22.5–50)	0.0001
e’ (cm/s)	6.1 (4.9–7.3)	8.1 (7.4–9.4)	5.9 (5–6.7)	5.0 (4.0–6.0)	5.1 (4.4–6.2)	0.0001
E/e’ ratio	12.6 (9.6–17.1)	9.1 (7.7–11.1)	11 (9.3–12.1)	17.9 (15.8–22.8)	23.1 (18.0–27.6)	0.0001
Septal thickness (cm)	1.2 (1.0–1.4)	1.1 (1.0–1.3)	1.2 (1.0–1.4)	1.2 (1.1–1.4)	1.1 (1.0–1.3)	0.0003
Posterior wall thickness (cm)	1.0 (0.9–1.2)	1.0 (0.8–1.1)	1.0 (0.9–1.2)	1.1 (0.9–1.2)	1.1 (1.0–1.3)	0.0001
End-diastolic diameter (cm)	4.6 (4.2–5.1)	4.6 (4.2–4.9)	4.6 (4.1–5.0)	4.7 (4.2–5.2)	5.4 (5.0–5.9)	0.0001
Prior MI	223 (38.7%)	44 (28%)	71 (40%)	92 (45%)	16 (40%)	0.01
Prior heart failure	228 (39.5%)	40 (26%)	57 (32%)	100 (49%)	31 (78%)	0.0001
Prior arrhythmia	24 (4.2%)	3 (2%)	8 (4%)	10 (5%)	3 (8%)	0.3
Diabetes	244 (42.3%)	47 (30%)	59 (33%)	116 (57%)	22 (55%)	0.0001
Hypertension	324 (56.1%)	77 (50%)	98 (55%)	126 (62%)	23 (58%)	0.1
Moderate or worse chronic lung disease	28 (4.9%)	5 (3%)	11 (6%)	10 (5%)	2 (5)	0.7
Peripheral vascular disease	67 (11.6%)	12 (8%)	14 (8%)	33 (16%)	8 (20%)	0.008
Creatinine	1.0 (0.9–1.3)	1.0 (0.9–1.2)	1.0 (0.9–1.2)	1.1 (0.9–1.3)	1.3 (1.0–2.3)	0.0001
Procedure						0.0001
CAB alone	446 (77.3%)	10 (6%)	19 (11%)	52 (26%)	13 (33%)	
AVR alone	94 (16.3%)	138 (89%)	153 (85%)	132 (65%)	23 (57%)	
CAB-AVR	37 (6.4%)	7 (5%)	7 (4%)	19 (9%)	4 (10%)	
Post-op creatinine	1.2 (1.0–1.6)	1.1 (1.0–1.4)	1.1 (1.0–1.4)	1.3 (1.0–1.9)	1.7 (1.2–2.9)	0.0001
Acute kidney injury (> 2× increase in creatinine)	35 (6%)	8 (5%)	7 (4%)	19 (9%)	1 (3%)	0.09
Length of hospital stay (days)	10 (8–15)	9 (7–13)	10 (8–14)	11 (7–17)	20.5 (12.5–24)	0.0001
Reintubation	16 (2.8%)	4 (3%)	5 (3%)	7 (3%)	0	0.7
ICU readmission	24 (4.2%)	7 (5%)	4 (2%)	10 (5%)	3 (8%)	0.4
Prolonged ventilation > 24 h	46 (8%)	6 (4%)	11 (6%)	22 (11%)	7 (18%)	0.009
AF	103 (17.9%)	29 (19%)	25 (14%)	39 (19%)	10 (25%)	0.3
In-hospital mortality	17 (3%)	3 (2%)	0	11 (5%)	3 (8%)	0.004

Data is displaced as median (interquartile range) for continuous variables and N(%) for categorical variables

Outcomes across grades of diastolic function are shown in the Table 1. Patients with more advanced DD had higher post-operative creatinine levels, longer hospital length of stay, higher rates of prolonged mechanical ventilation post-operatively, and higher rates of in hospital mortality. The patients who had AVR had higher rate of grade 2 or 3 diastolic dysfunction compared to those who underwent CABG alone (67.2% v. 34.8%, *p* < 0.001).

**Fig. 1:**
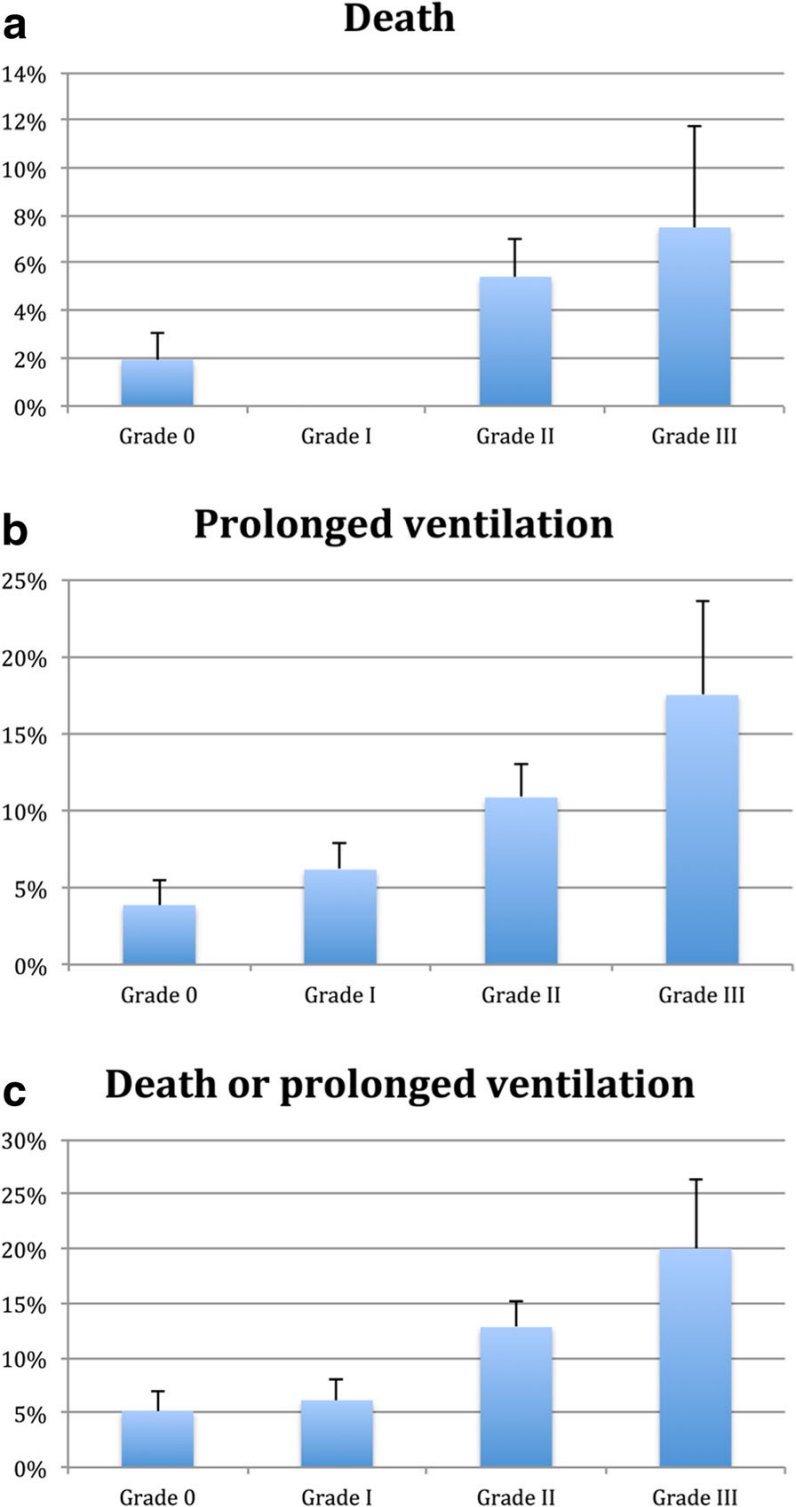
**a**-**c**: Increasing rates of death (*P* = 0.004), prolonged ventilation longer than 24 h (*P* = 0.009) and the composite of death and prolonged ventilation (*P* = 0.003) across grades of diastolic function

**Fig. 2:**
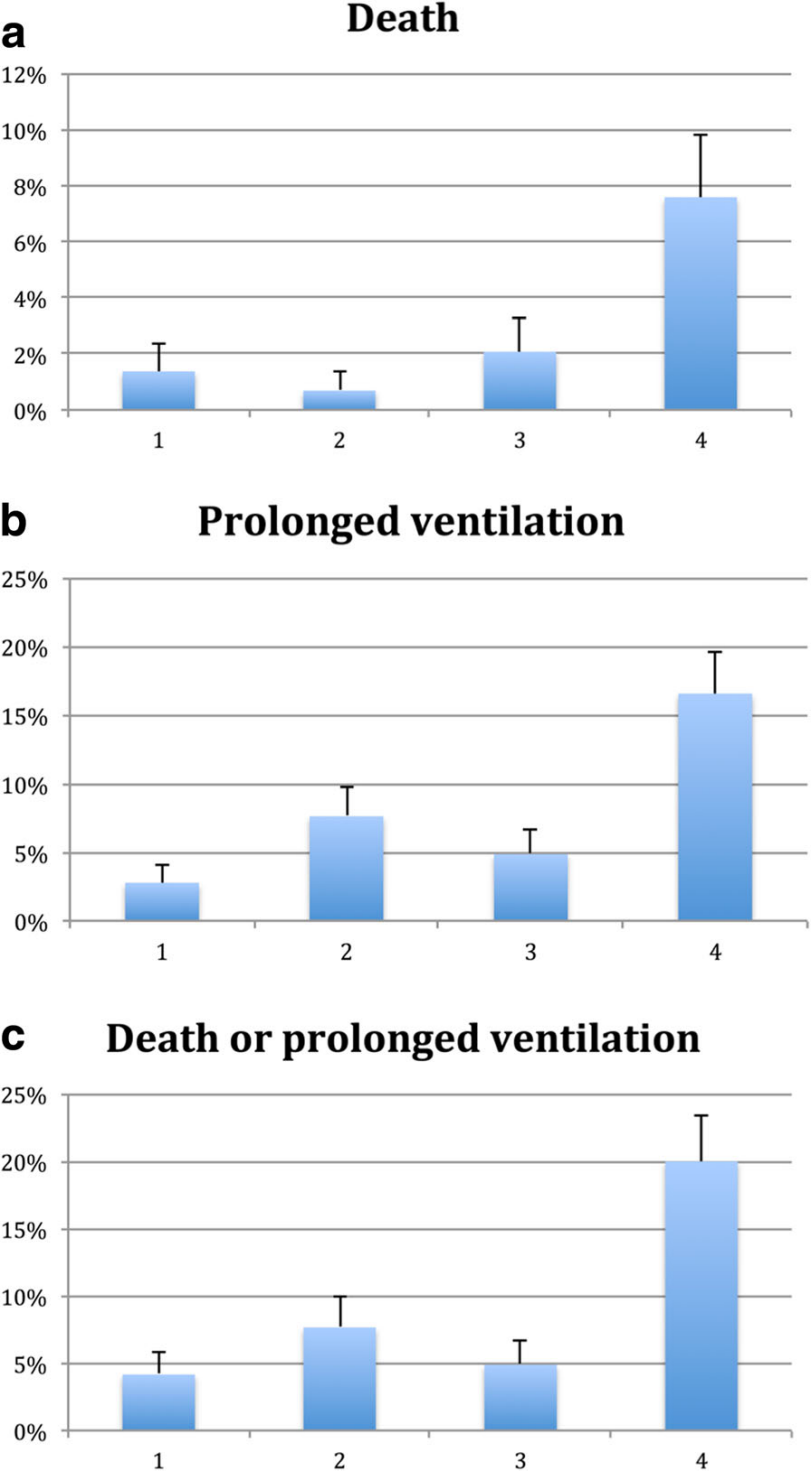
**a**-**c**: Increasing rates of death (*P* = 0.002), prolonged ventilation longer than 24 h (*P* = 0.001) and the composite of death and prolonged ventilation (*P* = 0.0001) across quartiles of increasing LV filling pressure, estimated by the E/e’ ratio

Figure 1 displays increasing rates of mortality, prolonged ventilation, and a composite of mortality and prolonged ventilation across increasing grade of DD. Rates of death and prolonged ventilation also increased across quartiles of increasing LV filling pressure, assessed by the E/e’ ratio, as shown in Fig. 2. The median E/e’ in quartile 1 was 8.2 (interquartile range (IQR) 7.1–8.9), quartile 2 was 11.1 (IQR 10.4–11.6), quartile 3 was 14.7 (IQR 13.7–15.9) and quartile 4 was 22.7 (IQR 19.5–27.6). There were increasing adverse events with increasing E/e’ ratio, with highest event rates in the highest quartile of E/e’, corresponding to an E/e’ ratio of 15 or higher. Diastolic dysfunction grade 2 or 3 remained associated with the composite of death or prolonged mechanical ventilation when the cohort was stratified by CABG alone versus AVR. In the CABG alone group, 5.8% of the group with diastolic function grade 0 or 1 suffered the composite endpoint compared to 11.6% of those with Diastolic dysfunction grade 2 or 3 (*p* = 0.031). In the AVR group, 4.7% of the group with diastolic function grade 0 or 1 suffered the composite endpoint compared to 18.2% of those with Diastolic dysfunction grade 2 or 3 (*p* = 0.035).

**Fig. 3:**
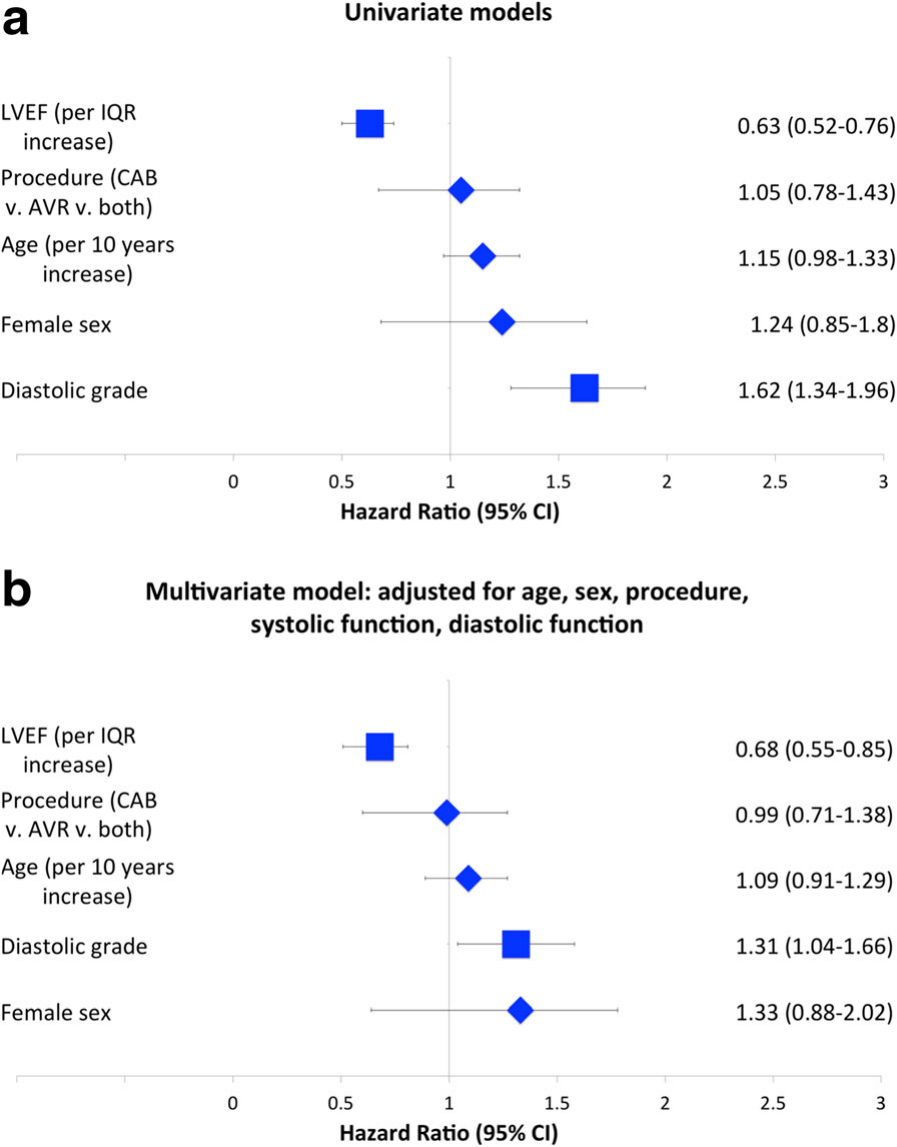
Univariate (Panel **a**) and multivariate (Panel **b**) logistic regression models for associations with the composite endpoint of death, prolonged mechanical ventilation, ICU readmission, and hospital length of stay longer than 14 days

Univariate logistic regression models for associations with the composite endpoint of death, prolonged mechanical ventilation, ICU readmission, and hospital length of stay longer than 14 days are shown in Fig. 3, panel A. Both systolic function (odds ratio 0.63 95% CI 0.52–0.76 per inter-quartile increase in LVEF) and diastolic function (odds ratio 1.62 95% CI 1.34–1.96 per increasing DD grade) were associated with outcome in univariate models. A multivariable model is shown in Fig. 3, B. In the model including age, sex, procedure, systolic and diastolic function, both systolic (odds ratio 0.68 95% CI 0.55–0.85 per inter-quartile increase in LVEF) and diastolic function (odds ratio 1.31 95% CI 1.04–1.66 per increasing DD grade) independently predicted outcome. Similar findings were obtained in considering quartile of E/e’ ratio: increasing quartile of E/e’ ratio was associated with the composite outcome in a univariate model (odds ratio 1.61 95% CI 1.34–1.92 per inter-quartile increased in E/e’ ratio) and the model adjusted for age, sex, procedure, and systolic function (odds ratio 1.40 95% CI 1.14–1.72 per inter-quartile increase in E/e’ ratio).

## Discussion

In a cohort of 577 patients undergoing cardiac surgery, we report the association of echocardiographic diastolic function with outcome. Our major findings include 1) DD is common among patients undergoing CAB, AVR or both, 2) higher grade of DD and higher LV filling pressure assessed by echocardiography is associated with higher risk of mortality and need for prolonged ventilation and 3) both systolic and diastolic function independently predict outcome after cardiac surgery.

We report that DD is common among patients undergoing cardiac surgery, with 42% of patients manifesting grade II or higher DD. These findings are consistent with community based cohorts, demonstrating abnormal diastolic function in between 27 and 56% of a community based sample [19, 20] and more common still among patients with underlying conditions also associated with cardiac surgical disease such as age, diabetes, renal insufficiency, coronary disease hypertension, and obesity [19, 21, 22]. Conditions that cause left ventricular hypertrophy, such as aortic stenosis and systemic hypertension, contribute uniquely to DD. Increased LV wall thickness and LV mass contribute to diastolic dysfunction by decreasing ventricular compliance and increasing left ventricular stiffness. These alterations contribute to changes in both the passive and active filling properties of the ventricle [1]. Furthermore, as the LV wall becomes thicker, there is regional dyssynchrony in contraction that can be observed which further contributes to diastolic dysfunction [23]. Given that DD is common in cardiac surgical patients, has prognostic value, and can be impacted by specific treatments as discussed below, consideration should be given to screening for DD prior to cardiac surgery.

We demonstrate that DD is associated with prolonged ventilation, death, and longer ICU and hospital length of stay. Other studies have also correlated diastolic grade with mortality [11, 13] and adverse events [15] after cardiac surgery, and the ratio of E to e’ which is a surrogate for LV filling pressure has also been shown to predict postoperative morbidity [12]. The unifying feature responsible for this adverse prognosis may be elevated left heart filling pressure which has itself been associated with mortality [24] and length of stay after surgery [25]. Other putative mechanisms of adverse prognosis include DD predisposing to atrial fibrillation, which has been suggested in some [9] but not all [10] studies. DD has been shown to correlate with difficulty separating from cardiopulmonary bypass [14] and to be associated with postoperative pulmonary edema which could explain need for prolonged ventilation [17].

We report that echocardiographic systolic and DD both predict outcome independently. While systolic function is commonly utilized as a risk marker prior to cardiac surgery [13, 26, 27], less attention has been paid to DD. DD has been shown to independently improve prognostic ability when considered with systolic dysfunction, improving model performance by 5.9% above that provided by the Society of Thoracic Surgeons’ risk score alone [13]. If future studies confirm our findings, an assessment of diastolic function or left heart filling pressure could be used to further refine prognosis prior to surgery or to delay surgery until diastolic indices improve after change in loading conditions or volume status [4, 18] or revascularization after myocardial infarction [8].

Although there are no treatments targeted specifically at the cellular perturbations underlying DD [3], several clinical parameters can be modified to improve diastolic function. Intra-aortic balloon pumping can improve diastolic function [28] and high levels of positive end-expiratory pressure on the ventilator can influence diastolic function by changing preload sensitivity [6, 7] and exacerbating defects in myocardial relaxation [5]. The calcium sensitizing agent levosimendan improves diastolic function at time of CABG by decreasing relaxation time and deceleration time, suggesting improved myocardial relaxation, and nitrate infusions have similar albeit less pronounced effects [29]. Control of heart rate is also important in patients with DD. Those patients with restrictive myocardial filling complete LV filling in early diastole and are thus “heart rate dependent,” as stroke volume is fixed and longer diastole will not improve filling [1, 2]. Such patients would benefit from permissively higher heart rates [30]. In contrast, those patients with an impaired relaxation filling pattern- grade I DD- benefit from longer filling periods and slower heart rates due to the prolonged myocardial relaxation time. Thus, once DD is identified prior to cardiac surgery, there are several therapeutic implications. Whether treating DD in this manner or via techniques or agents to be developed improves surgical outcomes should be a major focus of future research.

Limitations of our study include its retrospective, observational nature. Thus, we can assess for associations but cannot infer causality of DD and post-surgical outcomes. Our study focuses only on those patients in sinus rhythm in whom diastolic function can be graded, however patients with paced rhythms, mitral valve disease, and mitral annular calcification, and arrhythmia such as atrial fibrillation are major subsets of the cardiac surgical population; thus, although diastolic function cannot always be graded in these populations, markers of left ventricular filling pressure such as E/e’ ratio should be used. Our data set did not include details on completeness of revascularization and detailed conduct of cardiopulmonary bypass, and the association of diastolic function with these parameters needs to be more fully characterized in future studies. Finally, not all patients in practice will have adequate transthoracic echocardiographic windows and image quality for assessment of diastolic function, and correlation of our findings with those on intra-operative trans-esophageal echocardiography would be useful.

## Conclusions

In conclusion, DD is common among patients undergoing CAB, AVR and CAB-AVR and is associated with death, prolonged mechanical ventilation, and prolonged hospital and ICU length of stay independent of systolic dysfunction. Future studies should assess treatments targeted at improving diastolic function in cardiac surgical patients and the impact of therapy on post-operative outcomes.

## Abbreviations

AVR: Aortic valve replacement
CABG: Coronary artery bypass grafting
CI: Confidence interval
DD: Diastolic dysfunction
HFPEF: Heart failure with preserved ejection fraction
IQR: Interquartile range
LVEF: Left ventricular ejection fraction
TDI: Tissue Doppler imaging
TTE: Transthoracic echocardiography
